# Supplementary material for: Competitive Dynamics on Complex Networks
Source: Sci Rep. 2014 Jul 28;4:5858. doi: 10.1038/srep05858 (PMC5376163; doi:10.1038/srep05858)
Supplement: Supplementary Information [file srep05858-s1.pdf]

# Competitive Dynamics on Complex Networks

## Supplementary Information

Jiuhua Zhao, Qipeng Liu & Xiaofan Wang

Department of Automation, Shanghai Jiao Tong University, and Key Laboratory of System Control and Information Processing, Ministry of Education of China, Shanghai 200240, China.

## SUPPLEMENTARY FIGURE

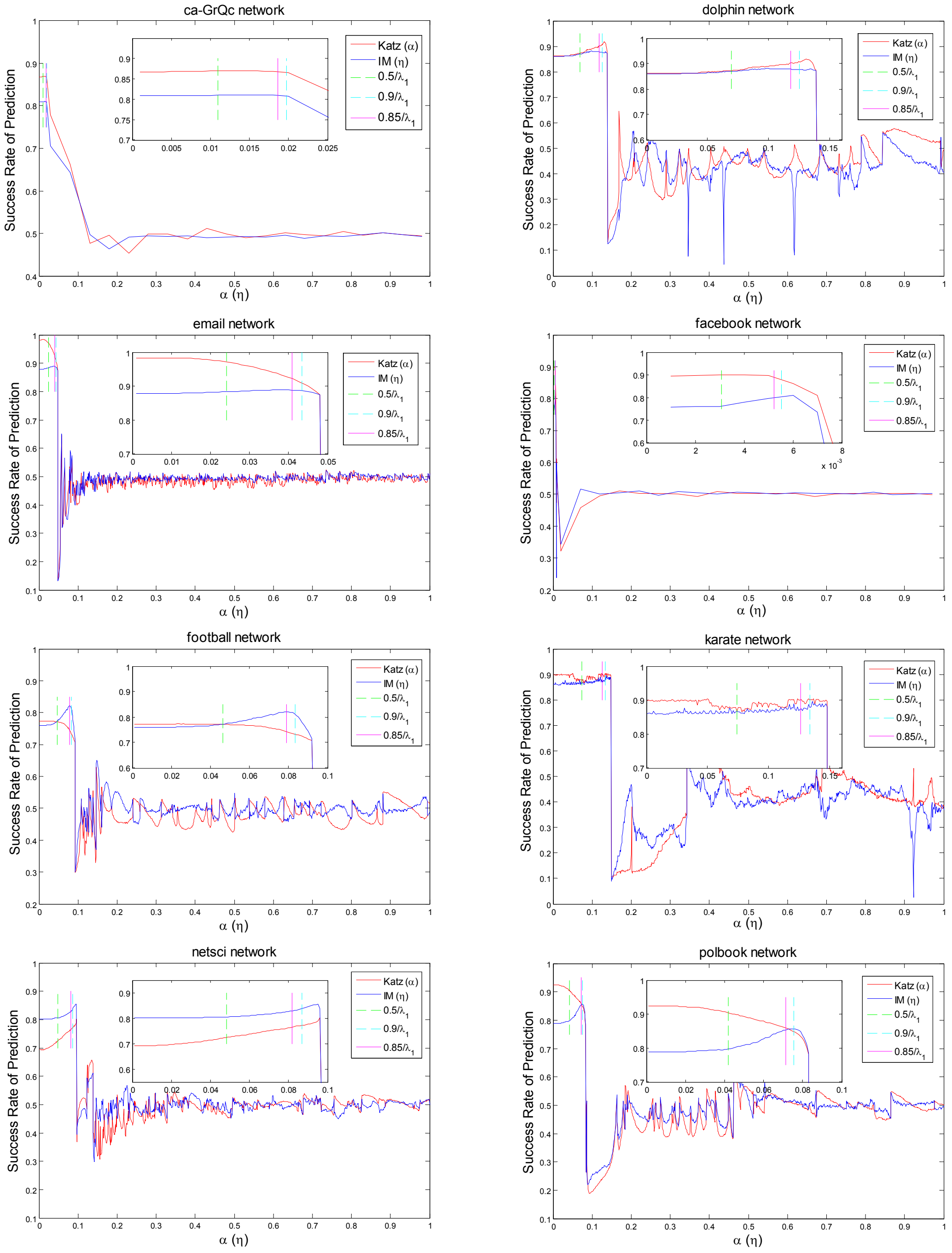

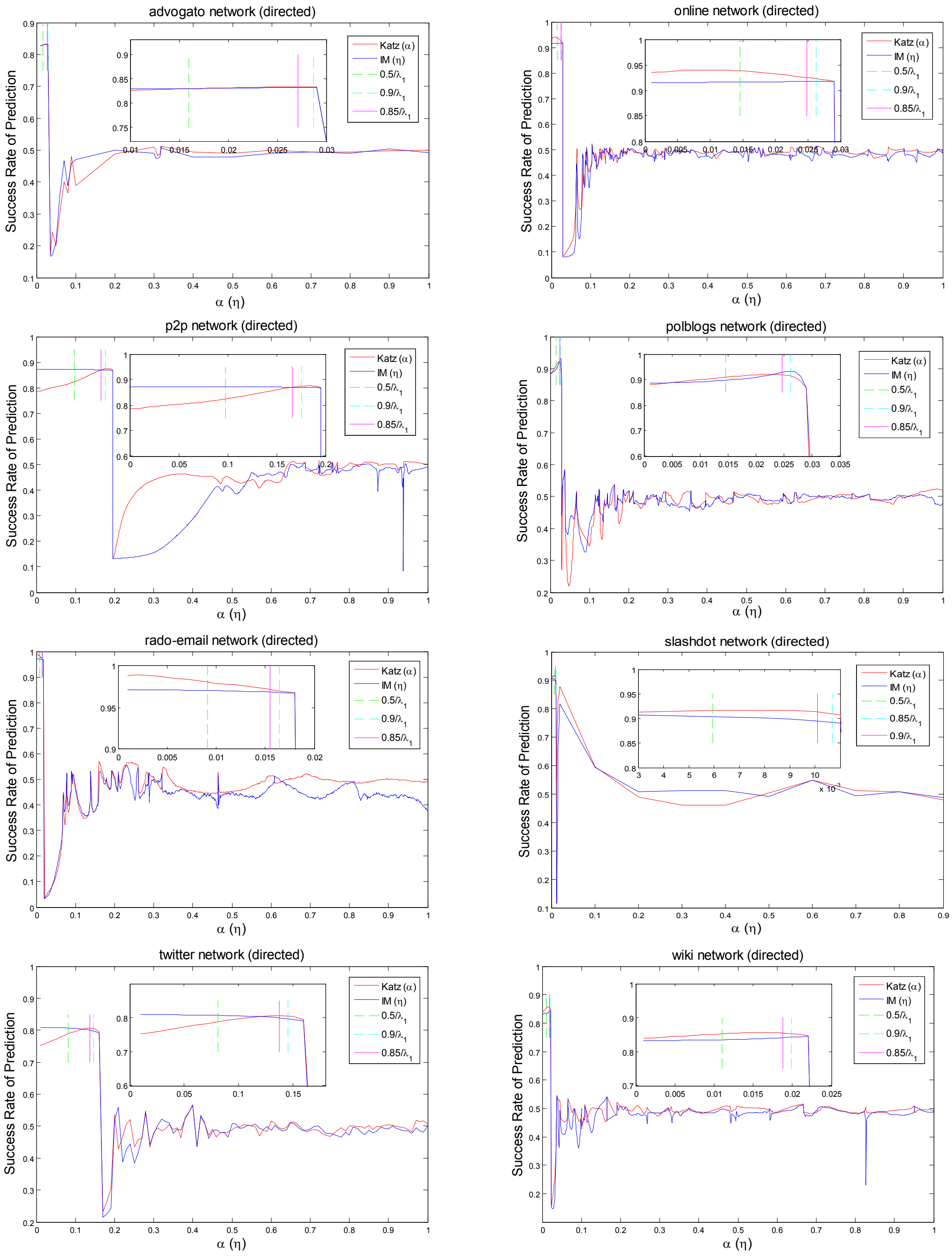


**Figure S1 | Choice of $\alpha$ and $\eta$ in 16 real networks.** As $\alpha$ is in the interval of $[0.5\lambda_1^{-1}, 0.9\lambda_1^{-1})$, the Katz Centrality has a relatively steady value, and correspondingly, the successful rate of prediction of the result of competition based on Katz (the red line) is almost unchanged for most networks. Meanwhile, it is found in simulations that the successful rate of prediction based on IM (the blue line) is also relatively high and robust as $\eta$ is in this interval.

To explain why a fraction of prediction of agents' biases goes wrong according to the IM criterion, we compute

$$\beta_{ij} \triangleq \frac{\max\limits_{k \in W}\left(f_{ki} - f_{kj}\right)}{\max\limits_{i,j}\left(\max\limits_{k \in V/\{i,j\}}\left(f_{kj} - f_{kj}\right)\right)}, \tag{S1}$$

where $W = \{k \mid \mathrm{sgn}(f_{ki} - f_{kj}) \neq \mathrm{sgn}(\overline{x}_k)\}$ is the set of incorrectly predicted agents in a network. We compute $\beta_{ij}$ for each pair of competitors and plot its distribution for every network.

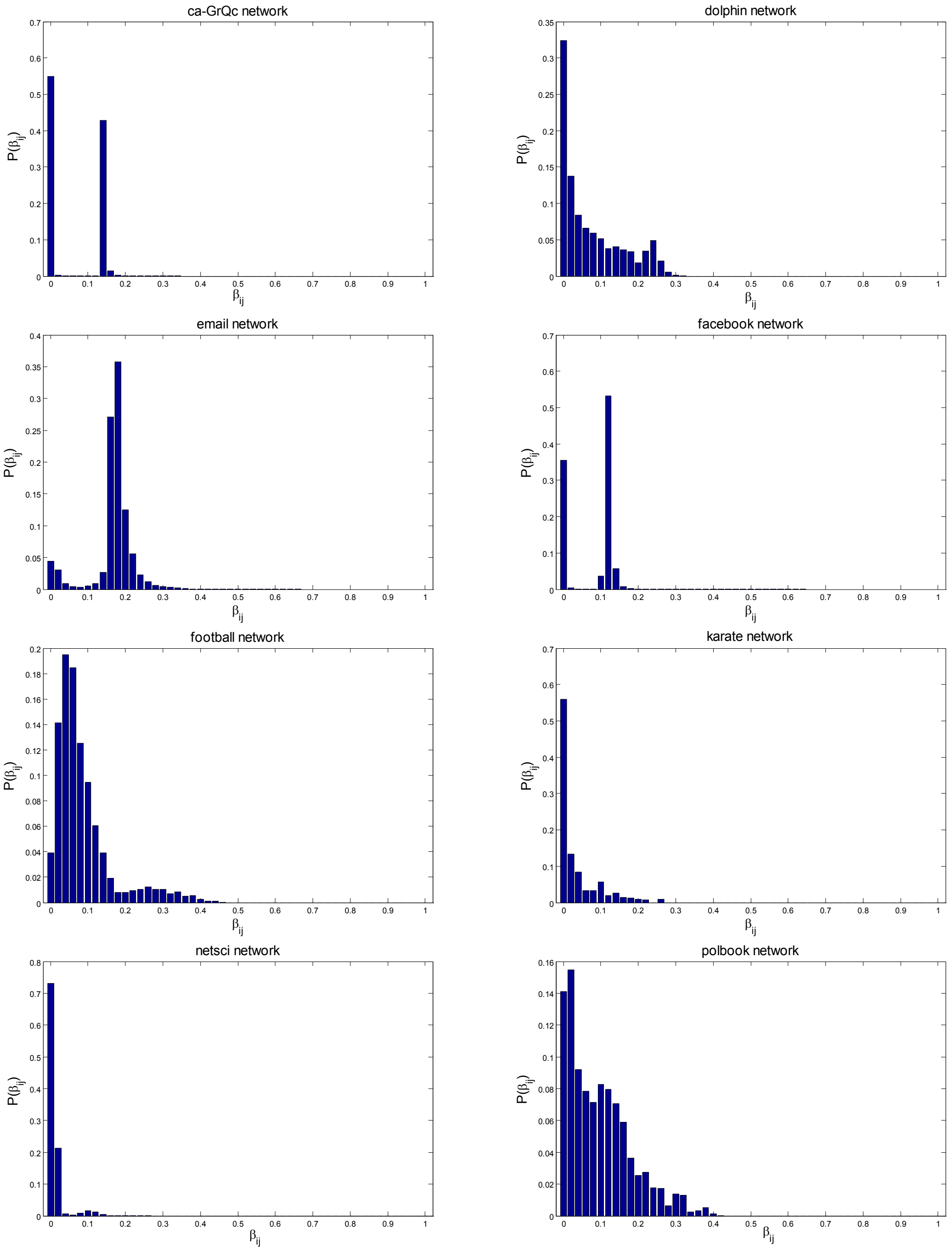

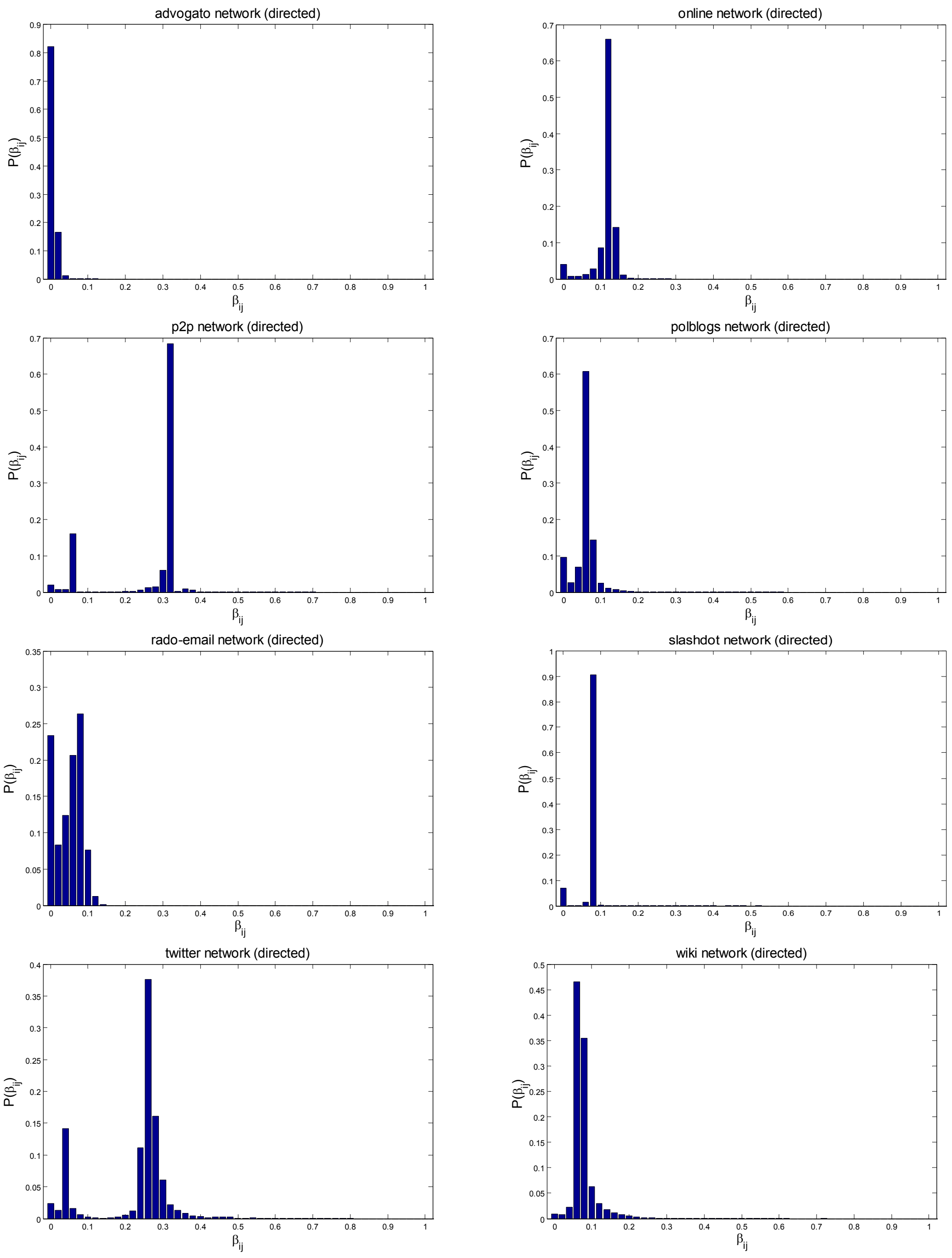


**Figure S2 | The distribution of $\beta_{ij}$ in 16 real networks.** For any given pair of agents $i$ and $j$, we can obtain a $\beta_{ij}$. In this way, we get the set $\beta = \{\beta_{ij}, \forall i \neq j \in V\}$ corresponding to all pairs of agents in the network and draw its distribution. Horizontal axis shows the value of $\beta_{ij}$ and vertical axis shows the proportion of the value. Values of $\beta_{ij}$ mainly distribute in the interval of [0, 0.4], which implies that incorrect predictions mainly correspond to small values of $\beta_{ij}$, i.e., for most pairs of competitors the prediction of a normal agent's bias based on IM criterion being incorrect is because two competitors have very similar influence on the normal agent.
